# Supplementary material for: Global evaluation of current and future threats to drylands and their vertebrate biodiversity
Source: Nat Ecol Evol. 2024 Jul 4;8(8):1448–58. doi: 10.1038/s41559-024-02450-4 (PMC11310083; doi:10.1038/s41559-024-02450-4)
Supplement: Supplementary file 1 — Supplementary Figs. 1–5. [file 41559_2024_2450_MOESM1_ESM.pdf]

nature ecology & evolution

Supplementary information https://doi.org/10.1038/s41559-024-02450-4

Global evaluation of current and future threats to drylands and their vertebrate biodiversity

In the format provided by the authors and unedited

Supplementary Information:

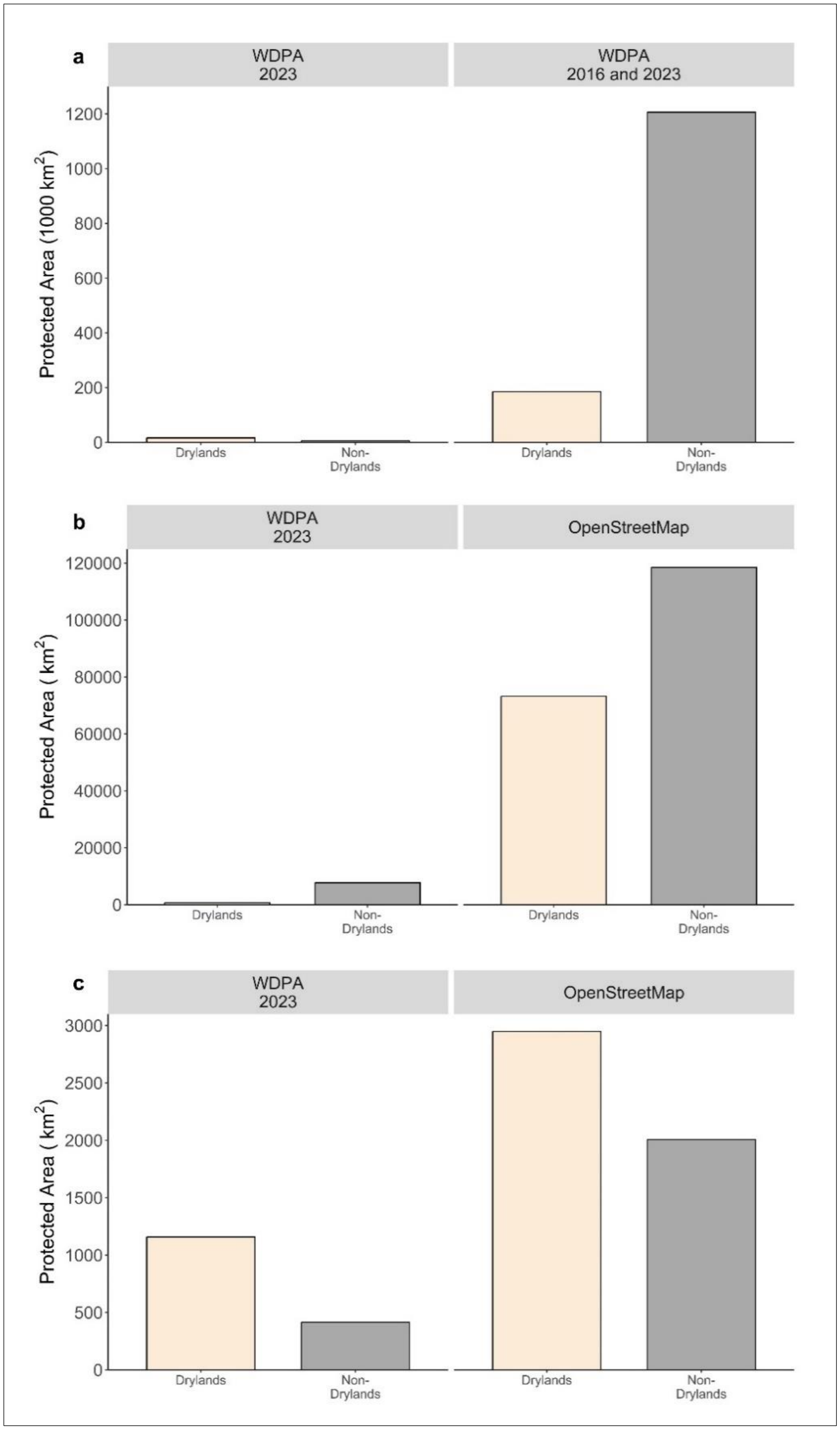

Supplementary Figure 1. Protected areas for regions lacking updated World Database on Protected Areas (WDPA) data: China (a), India (b), and Turkey (c). IUCN categories I-VI and uncategorized (i.e., 'Not Applicable', 'Not Assigned' and 'Not Reported').

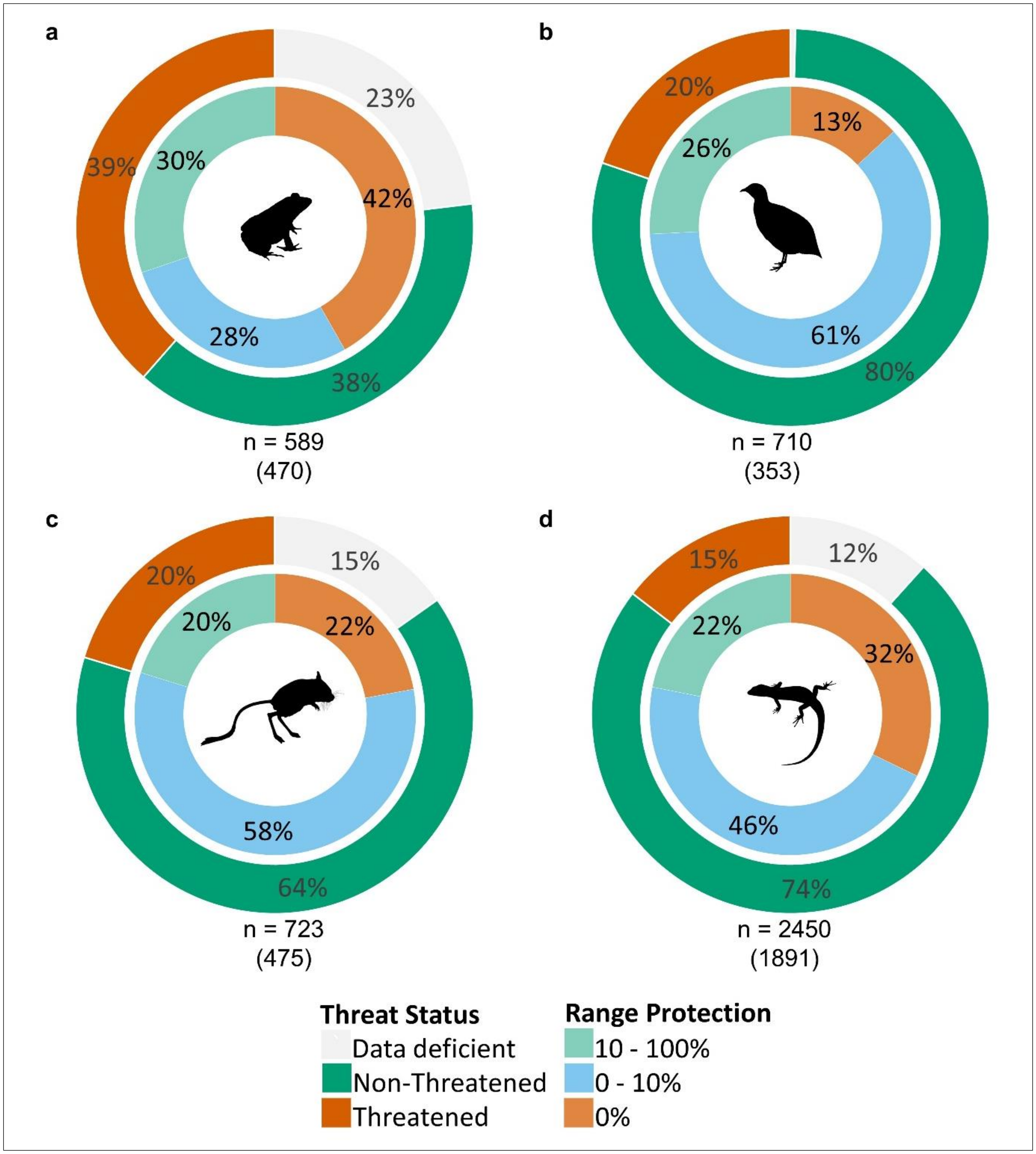

Supplementary Figure 2. Proportion range size protected and IUCN threat status of amphibian (a), bird (b), mammal (c), and reptile (d) species in drylands (≥90% global range in drylands). Inner circles show proportion of species with range size protected (IUCN categories I-IV): orange = 0% range protected, blue = 0-10% range protected, green = 10-100% range protected. Outer circles show proportion of IUCN threatened species: dark orange = threatened (IUCN categories CR, EN, VU), dark green = non-threatened (IUCN categories NT, LC), grey = data deficient (IUCN category DD). n = number of species, parentheses = number of endemic species (>99% range in drylands).

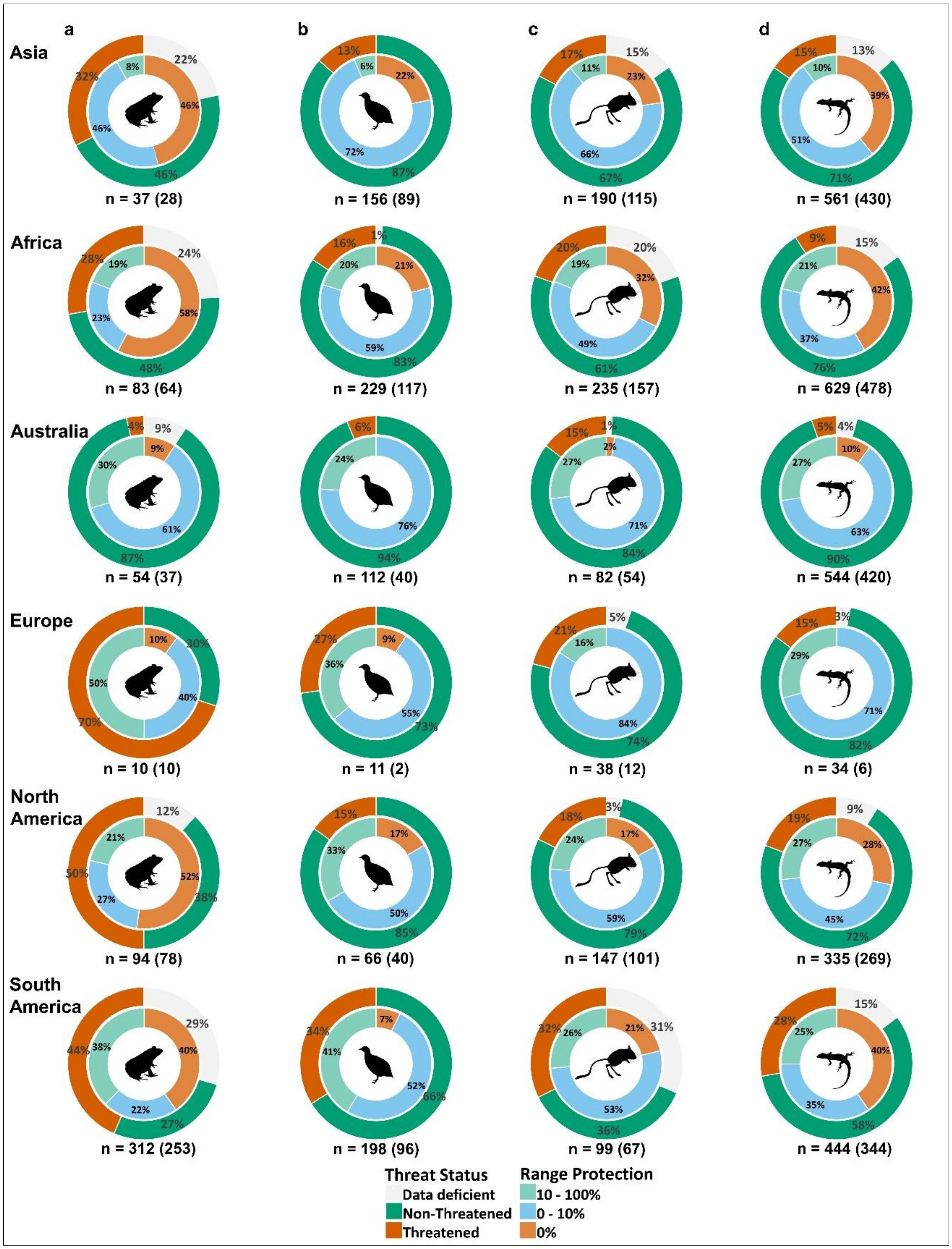

Supplementary Figure 3. Proportion range size protected and IUCN threat status of amphibian (a), bird (b), mammal (c), and reptile (d) species by continent in drylands (≥90% global range in drylands). Inner circles show proportion of species with range size protected (IUCN categories I-IV): orange = 0% range protected, blue = 0-10% range protected, green = 10-100% range protected. Outer circles show proportion of IUCN threatened species: dark orange = threatened (IUCN categories CR, EN, VU), dark green = non-threatened (IUCN categories NT, LC), grey = data deficient (IUCN category DD). n = number of species, parentheses = number of endemic species (>99% range in drylands).

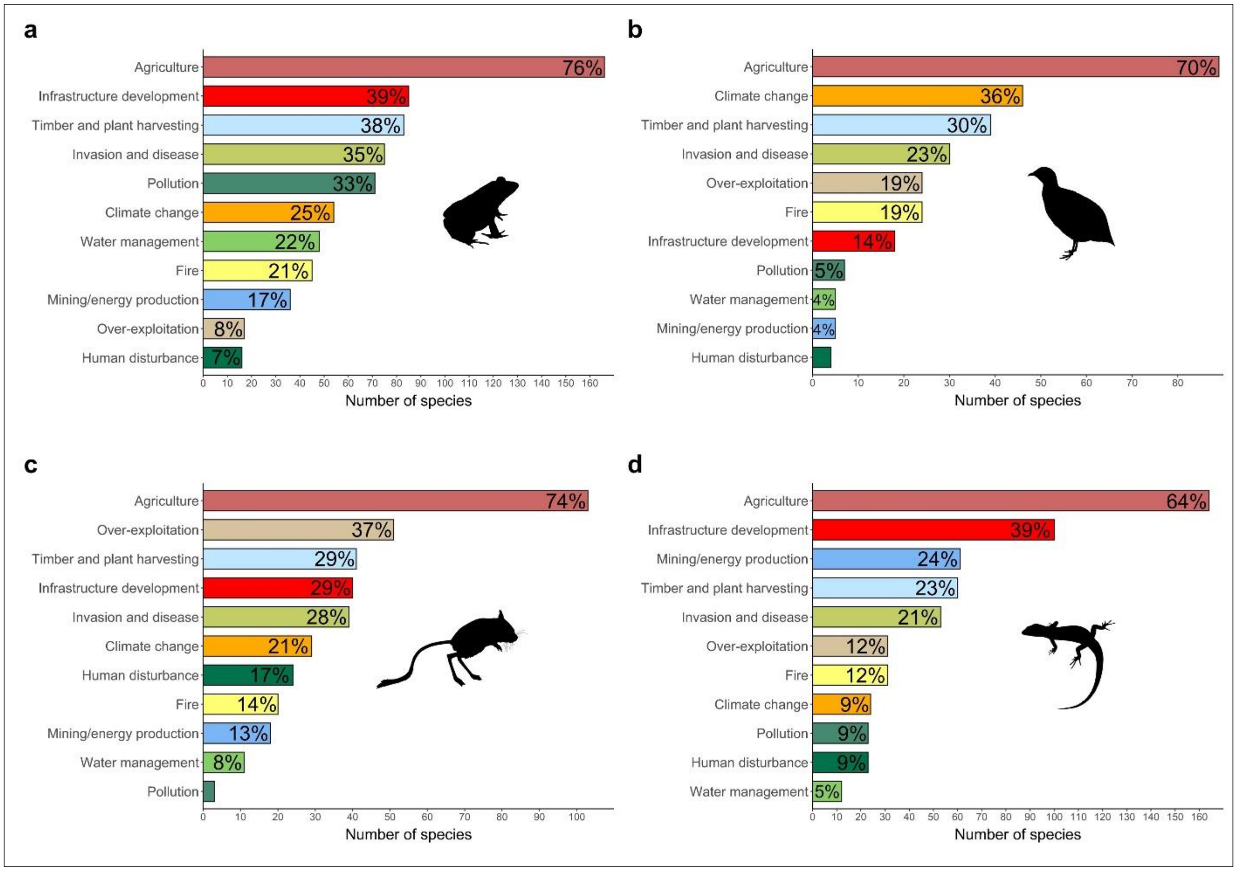

Supplementary Figure 4. Types of threats affecting amphibian (a), bird (b), mammal (c), and reptile (d) threatened species (in IUCN categories CR, EN, VU) in drylands (≥90% global range in drylands). Percentages show the proportion of species assessed affected by threats per taxon. Most species are subjected to multiple threats.

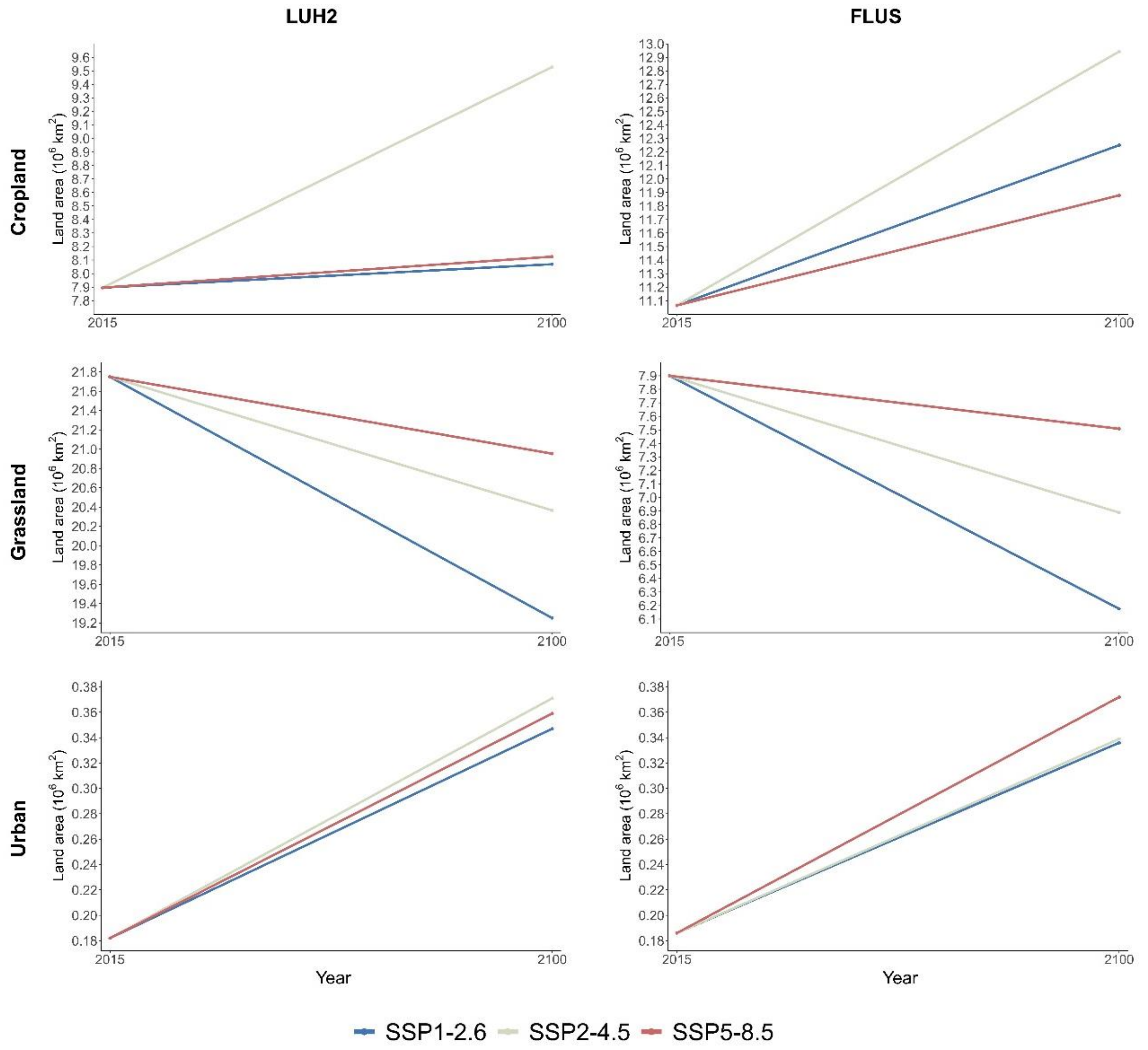

Supplementary Figure 5. Plots showing changes in land area for major land types in drylands for datasets LUH2 and FLUS (from Chen et al., 2022)[78] under SSP scenarios, showing comparable trends for the major land types considered. Lines show differences in land area (10^6 km) between years 2015 and 2100 for scenario SSP1-2.6 (2100) in blue, SSP2-4.5 (2100) in green, and SSP5-8.5 (2100) in red.
