# Supplementary material for: Global evaluation of current and future threats to drylands and their vertebrate biodiversity
Source: Nat Ecol Evol. 2024 Jul 4;8(8):1448–58. doi: 10.1038/s41559-024-02450-4 (PMC11310083; doi:10.1038/s41559-024-02450-4)
Supplement: Supplementary file 2 — Reporting Summary [file 41559_2024_2450_MOESM2_ESM.pdf]

nature portfolio

Corresponding author(s): Amir Lewin

Last updated by author(s): Feb 5, 2024

Reporting Summary

Nature Portfolio wishes to improve the reproducibility of the work that we publish. This form provides structure for consistency and transparency in reporting. For further information on Nature Portfolio policies, see our Editorial Policies and the Editorial Policy Checklist.

Statistics

For all statistical analyses, confirm that the following items are present in the figure legend, table legend, main text, or Methods section.

n/a	Confirmed	
☒	☐	The exact sample size (*n*) for each experimental group/condition, given as a discrete number and unit of measurement
☒	☐	A statement on whether measurements were taken from distinct samples or whether the same sample was measured repeatedly
☒	☐	The statistical test(s) used AND whether they are one- or two-sided<br>*Only common tests should be described solely by name; describe more complex techniques in the Methods section.*
☒	☐	A description of all covariates tested
☒	☐	A description of any assumptions or corrections, such as tests of normality and adjustment for multiple comparisons
☒	☐	A full description of the statistical parameters including central tendency (e.g. means) or other basic estimates (e.g. regression coefficient) AND variation (e.g. standard deviation) or associated estimates of uncertainty (e.g. confidence intervals)
☒	☐	For null hypothesis testing, the test statistic (e.g. *F*, *t*, *r*) with confidence intervals, effect sizes, degrees of freedom and *P* value noted<br>*Give P values as exact values whenever suitable.*
☒	☐	For Bayesian analysis, information on the choice of priors and Markov chain Monte Carlo settings
☒	☐	For hierarchical and complex designs, identification of the appropriate level for tests and full reporting of outcomes
☒	☐	Estimates of effect sizes (e.g. Cohen's *d*, Pearson's *r*), indicating how they were calculated

Our web collection on statistics for biologists contains articles on many of the points above.

Software and code

Policy information about availability of computer code

Data collection	No software was used for data collection.
Data analysis	Spatial analyses were performed in ArcGIS (v10.8.1.)<br>Data analysis was performed in R version 4.2.2 using the following packages:<br>wdpar package and the function 'wdpa_clean'

For manuscripts utilizing custom algorithms or software that are central to the research but not yet described in published literature, software must be made available to editors and reviewers. We strongly encourage code deposition in a community repository (e.g. GitHub). See the Nature Portfolio guidelines for submitting code & software for further information.

Data

Policy information about availability of data

All manuscripts must include a data availability statement. This statement should provide the following information, where applicable:

- Accession codes, unique identifiers, or web links for publicly available datasets
- A description of any restrictions on data availability
- For clinical datasets or third party data, please ensure that the statement adheres to our policy

The Drylands Datatset (2007) was obtained from UNEP-WCMC (https://datadownload.unep-wcmc.org/datasets).
Protected area coverage data were obtained from the World Database on Protected Areas (www.protectedplanet.net/), and OpenStreetMap (from

www.openstreetmap.org/).
Species distribution data are available for amphibians and mammals from the IUCN (v.6.3; www.iucnredlist.org/), birds from BirdLife International (v.4; http://datazone.birdlife.org/), and reptiles from the Global Assessment of Reptile Distributions (GARD v.1.7).
IUCN Red List data and IUCN threat-classifications were obtained from the IUCN (www.iucnredlist.org).
Land use data was obtained from the Land Use Harmonization (LUH2) project (v2h) from the year 2015 (http://luh.umd.edu/). Future land use data was obtained from phase 6 of the Coupled Model Intercomparison Project (CMIP6) from LUH2 (http://luh.umd.edu/).

Research involving human participants, their data, or biological material

Policy information about studies with human participants or human data. See also policy information about sex, gender (identity/presentation), and sexual orientation and race, ethnicity and racism.

Reporting on sex and gender	Not applicable
Reporting on race, ethnicity, or other socially relevant groupings	Not applicable
Population characteristics	Not applicable
Recruitment	Not applicable
Ethics oversight	Not applicable

Note that full information on the approval of the study protocol must also be provided in the manuscript.

Field-specific reporting

Please select the one below that is the best fit for your research. If you are not sure, read the appropriate sections before making your selection.

☐ Life sciences ☐ Behavioural & social sciences ☒ Ecological, evolutionary & environmental sciences

For a reference copy of the document with all sections, see nature.com/documents/nr-reporting-summary-flat.pdf

Ecological, evolutionary & environmental sciences study design

All studies must disclose on these points even when the disclosure is negative.

Study description	A global assessment of the degree of protected area and threats to biodiversity in drylands due to current and future human-induced land-use changes
Research sample	See Data description above
Sampling strategy	All relevant data were used. No statistical methods were used to predetermine sample size
Data collection	Data were based on existing datasets and was collected online by the authors
Timing and spatial scale	Global data from 2015 to 2100
Data exclusions	No data were excluded from the analyses
Reproducibility	All data are accessible online
Randomization	Not applicable
Blinding	Not applicable

Did the study involve field work? ☐ Yes ☒ No

Reporting for specific materials, systems and methods

We require information from authors about some types of materials, experimental systems and methods used in many studies. Here, indicate whether each material, system or method listed is relevant to your study. If you are not sure if a list item applies to your research, read the appropriate section before selecting a response.

Materials & experimental systems

n/a	Involved in the study
☒	☐ Antibodies
☒	☐ Eukaryotic cell lines
☒	☐ Palaeontology and archaeology
☒	☐ Animals and other organisms
☒	☐ Clinical data
☒	☐ Dual use research of concern
☒	☐ Plants

Methods

n/a	Involved in the study
☒	☐ ChIP-seq
☒	☐ Flow cytometry
☒	☐ MRI-based neuroimaging

Plants

Seed stocks

Not applicable

Novel plant genotypes

Not applicable

Authentication

Not applicable
